# Immune signaling as a determinant of cellular identity and tissue function

**DOI:** 10.3389/fimmu.2026.1831391

**Published:** 2026-06-12

**Authors:** Rahul Mittal, Farhad Alipour, Abhi Dhote, Roshini Shivakumar, Khemraj Hirani

**Affiliations:** 1 Diabetes Research Institute, University of Miami Miller School of Medicine, Miami, FL, United States; 2 Division of Endocrinology, Diabetes, and Metabolism, Department of Medicine, University of Miami Miller School of Medicine, Miami, FL, United States

**Keywords:** cellular identity, cytokine networks, immune regulation, immune-driven reprogramming, immune-tissue crosstalk, inflammatory mediators, tissue function

## Abstract

Immunology has traditionally interpreted tissue pathology through models centered on immune-mediated cytotoxicity. In this prevailing model, functional decline is considered a downstream consequence of cell death and irreversible loss of cellular mass. Increasing evidence across metabolically active tissues challenges this paradigm by demonstrating that immune-derived signals exert potent regulatory effects on cellular identity that are independent of overt cytotoxicity. Cytokines, interferons, and inflammatory mediators directly engage transcriptional, epigenetic, and stress-response networks that sustain differentiated states. This engagement leads to suppression of lineage-defining gene programs, destabilization of identity-enforcing regulatory circuits, and sustained impairment of specialized function while cellular viability is preserved. In this Perspective, we propose a conceptual reframing in which immune systems are considered continuous regulators of cellular identity stability rather than solely as determinants of cell survival. The process becomes stabilized through epigenetic remodeling and generates persistent dysfunctional states that may be variably reversible depending on the duration and intensity of immune exposure. The proposed framework distinguishes immune-induced plasticity from epigenetically fixed identity failure. By repositioning immune instruction of cellular identity as one of the central mechanisms of disease pathogenesis, this framework opens new conceptual avenues. It further emphasizes therapeutic strategies focused on restoring tissue function through the reestablishment of differentiated states.

## Introduction

Immunology has long been grounded in a framework that emphasizes host defense, immune tolerance, and the cytotoxic elimination of infected, transformed, or damaged cells (1, 2). Within this paradigm, tissue pathology is most often interpreted as the downstream consequence of immune-mediated injury, in which inflammatory responses culminate in cell death and irreversible loss of functional mass (1, 3, 4). This framework has contributed to understanding mechanisms of pathogen clearance, autoimmunity, and transplant rejection, and it continues to shape prevailing models of immune involvement in chronic disease (5). However, when applied to metabolically active tissues that fail functionally before they are lost, this damage-centric approach is insufficient (6, 7). Cytokines, interferons, and other inflammatory mediators directly intersect with transcriptional networks, epigenetic regulators, and stress-response pathways that maintain differentiated identity (8). In multiple tissues, immune exposure induces suppression of lineage-defining gene programs, activation of alternative or progenitor-associated transcriptional states, and durable changes in chromatin organization, while cellular viability is preserved (6, 9, 10). These observations argue that the immune system shapes tissue function not only by determining cell survival but also by actively instructing cellular identity (10–12).

At a mechanistic level, immune signaling is initiated through tightly coordinated receptor-proximal and intracellular pathways that regulate cytokine biosynthesis, processing, and secretion in a context-dependent manner (1–3, 13–17) (**Table 1**). These pathways integrate inputs from pattern recognition receptors (PRRs), metabolic sensors, and stress-responsive signaling networks to ensure appropriate immune activation. Pro-inflammatory cytokines such as interleukin-1 beta (IL-1β) and interleukin-18 (IL-18) are synthesized as inactive precursors. They undergo inflammasome-dependent proteolytic maturation mediated by caspase-1 following activation of multiprotein complexes such as nucleotide-binding oligomerization domain-like receptor family pyrin domain-containing 3 (NLRP3) (8, 44–47). In contrast, cytokines such as tumor necrosis factor (TNF) and interleukin-6 (IL-6) are secreted through conventional endoplasmic reticulum–Golgi trafficking or through non-classical pathways involving secretory lysosomes, extracellular vesicles, and gasdermin-mediated pore formation. These tightly regulated processes act as critical regulatory nodes. They encode signal strength, duration, and spatial distribution of immune activity, thereby shaping the magnitude and tissue-specific spread of inflammation (48–50) (**Table 1**).

**Table 1:** Immune regulation of cellular identity: a multiscale mechanistic framework.

Level of regulation	Immune input characteristics	Core molecular processes	Primary regulatory substrates	Direction of regulatory change	Cellular identity outcome	Functional consequence	Reversibility profile	References
Receptor-proximal signaling	Acute cytokine exposure (TNF, IL-1β, IFN-γ); Toll-like receptor (TLR)-mediated sensing of pathogen- and damage-associated signals	Activation of NF-κB, JAK–STAT, IRF, MAPK cascades; MyD88- and TRIF-dependent TLR signaling pathways	Signal-dependent transcription factors and cofactors	Rapid redistribution of TF activity across target loci	Transient modulation of lineage-associated transcription	Reduced specialized gene expression with preserved core identity	High (signal withdrawal restores baseline)	(4, 18–22)
Transcriptional network remodeling	Sustained cytokine gradients; repeated stimulation	Altered TF occupancy, cofactor competition, and promoter–enhancer engagement	Promoters, enhancers, and super-enhancers	Selective attenuation of lineage-defining transcriptional programs	Partial identity shift with retention of lineage markers	Compromised functional output with maintained viability	Moderate (context-dependent recovery)	(23–25)
Epigenetic reprogramming	Chronic inflammatory signaling; persistent cytokine exposure	Histone modification (acetylation, methylation), chromatin remodeling, DNA methylation changes	Chromatin accessibility landscapes at regulatory elements	Stabilization of stress-responsive chromatin states	Reinforced alternative transcriptional configurations	Persistent suppression of specialized functions	Partial (requires active reprogramming)	(26–28)
3D genome reorganization	Prolonged inflammation; repeated immune activation cycles	Chromatin looping alterations; enhancer–promoter rewiring; compartment shifts	Higher-order chromatin architecture and regulatory hubs	Reconfiguration of long-range regulatory interactions	Coordinated restructuring of gene expression programs	Network-level dysregulation of functionally linked genes	Limited (structural constraints persist)	(29–34)
Metabolic–epigenetic coupling	Cytokine-driven metabolic stress; altered nutrient flux	Changes in glycolysis, TCA cycle, redox balance affecting cofactor availability (e.g., acetyl-CoA, α-KG, NAD^+^)	Chromatin-modifying enzyme activity dependent on metabolites	Biochemical constraint on chromatin modification dynamics	Bias toward transcriptional programs compatible with altered metabolism	Reduced biosynthetic and functional capacity	Context-dependent (metabolic restoration required)	(8, 35, 36)
Gene regulatory Network (GRN) stabilization	Recurrent or chronic immune signaling	Feedback reinforcement, network rewiring, and loss of regulatory flexibility	Integrated gene regulatory networks	Transition from dynamic to stabilized network states	Identity drift progressing toward fixed alternative states	Durable dysfunction without lineage conversion	Low (network-level constraints dominate)	(37–39)
Tissue-level integration	Persistent immune–parenchymal interaction; microenvironmental signaling	Cell–cell communication, cytokine gradients, stromal–immune coupling	Multicellular regulatory circuits	Propagation of identity changes across cell populations	Coordinated tissue-level identity remodeling	Organ-level functional decline without overt cell loss	Low to moderate (depends on tissue context)	(17, 40–43)

TLR, Toll-like receptor; PRR, pattern recognition receptor; TNF, tumor necrosis factor; IL, interleukin; IFN, interferon; NF-κB, nuclear factor kappa B; JAK–STAT, Janus kinase–signal transducer and activator of transcription; IRF, interferon regulatory factor; MAPK, mitogen-activated protein kinase; MyD88, myeloid differentiation primary response 88; TRIF, TIR-domain-containing adapter-inducing interferon-β; TF, transcription factor; GRN, gene regulatory network; TCA cycle, tricarboxylic acid cycle; α-KG, alpha-ketoglutarate; NAD^+^, nicotinamide adenine dinucleotide (oxidized form).

Importantly, the quantitative and qualitative features of these signaling dynamics influence how immune-derived cues are sensed by parenchymal cells. These features include cytokine gradients, temporal oscillations, and combinatorial signaling inputs. They are integrated into intracellular signaling networks that converge on transcriptional regulators and chromatin-modifying machinery (26, 51, 52). Through this integration, immune signals modulate the activity of transcription factors such as nuclear factor kappa B (NF-κB), signal transducer and activator of transcription proteins (STATs), and interferon regulatory factors (IRFs) (9–11, 53). They also engage epigenetic modifiers that regulate chromatin accessibility, histone modification states, and enhancer activity. Together, these processes reprogram gene regulatory networks that define and stabilize cellular identity across tissues (9–11, 53) (**Table 1**).

Beyond their classical roles in host defense and immune surveillance, immune systems are increasingly recognized as integral components of tissue homeostasis (16, 49, 54–56). Immune cells and their associated signaling networks are constitutively present within most organs, where they engage in continuous communication with parenchymal cells under physiological conditions (40, 48, 57, 58). These interactions are responsive to metabolic flux, mechanical strain, and cellular stress and occur independently of overt infection or injury (17, 59–61). As a result, immune signaling functions not only as a reactive system but also as a persistent contextual input that influences how tissues maintain function and adapt to environmental and physiological demands (62–65) (**Table 1**).

This expanded view of immune-tissue interaction suggests that immune-derived cues can exert sustained effects on the cellular state over time (66–68). As differentiated function requires continuous reinforcement of transcriptional and regulatory programs, chronic exposure to immune signals has the potential to modulate cellular competence without inducing cell death (69–71). In this framework, immune activity can progressively reshape the molecular architecture that supports specialized function, leading to functional decline that precedes irreversible structural loss (72–74). Recognizing immune signaling as a regulator of the cellular state provides a foundation for understanding how chronic inflammatory environments give rise to durable tissue dysfunction in the absence of extensive cytotoxic injury (75–78).

**Figure 1:**
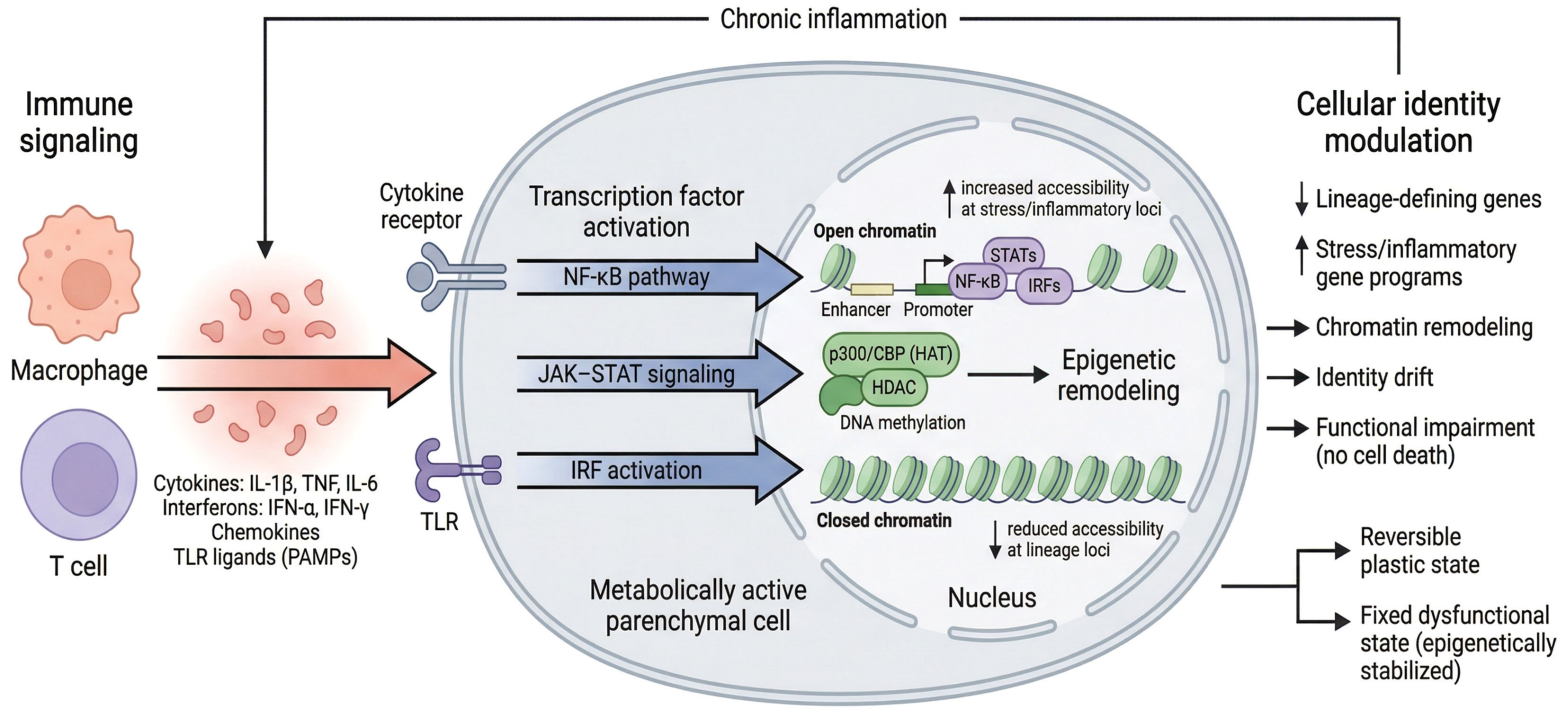
Immune signaling regulates cellular identity and function. Immune-derived factors from macrophages and T cells, including cytokines, interferons, chemokines, and TLR ligands, activate NF-κB, JAK–STAT, and IRF pathways in parenchymal cells. These signals drive transcription factor activation and recruitment of chromatin-modifying complexes, leading to epigenetic remodeling and altered chromatin accessibility. Chronic inflammatory signaling promotes activation of stress-responsive gene programs and suppression of lineage-defining genes, resulting in identity drift and functional impairment without cell death. Depending on exposure dynamics, cells may remain in a reversible plastic state or progress to a stabilized dysfunctional state. This framework highlights immune signaling as a continuous regulator of cellular identity. Created in BioRender. Mittal, R. (2026) https://BioRender.com/7efxg6z NF-κB, nuclear factor kappa B; JAK–STAT, Janus kinase–signal transducer and activator of transcription; IRF, interferon regulatory factor; TLR, Toll-like receptor; IL-1β, interleukin-1 beta; TNF, tumor necrosis factor; IL-6, interleukin-6; IFN-α, interferon alpha; IFN-γ, interferon gamma; PAMPs, pathogen-associated molecular patterns; HDAC, histone deacetylase.

In this Perspective, we propose a conceptual reframing of immune–tissue interactions in which immune systems can be considered as active arbiters of cellular identity stability rather than as episodic mediators of injury (**Figure 1**). Within this framework, immune-derived signals function as continuous regulatory inputs that shape the maintenance, robustness, and limits of differentiated cellular states by influencing the transcriptional and epigenetic architectures that sustain specialization (**Figure 1**). These signals operate across physiological and pathological contexts to calibrate functional competence, govern adaptive responses to stress, and bias cells toward trajectories of stability, plasticity, or dysfunction. By positioning immune signaling as a determinant of identity maintenance over time, this model provides a mechanistic basis for explaining how tissue dysfunction can arise progressively and persist independently of overt cell loss. The significance of this framework lies in its ability to unify diverse observations across chronic inflammatory diseases under a common principle of immune-instructed remodeling of cellular state. By reframing tissue dysfunction as a consequence of altered identity regulation rather than irreversible damage, it provides a revised mechanistic understanding of disease pathogenesis. This framework has direct therapeutic implications by shifting emphasis towards the restoration of the differentiated function through modulation of identity programs.

To provide mechanistic specificity, cellular identity can be formally defined as a metastable attractor state within a multidimensional gene regulatory landscape, maintained through continuous reinforcement by lineage-restricted transcription factor networks, chromatin accessibility constraints, and metabolic coupling (79–81) (**Table 1**). Immune-derived signals interface with this regulatory architecture across distinct but interdependent layers (82–84). At the signaling level, pathway-specific activation of NF-κB, JAK–STAT, and IRF modules encodes stimulus-dependent information through combinatorial and temporal dynamics (85–89). At the regulatory level, these signals modulate transcription factor occupancy, cofactor redistribution, and enhancer selection, thereby altering the topology of active gene regulatory circuits (90–93). At the epigenetic level, signal-dependent recruitment of chromatin-modifying complexes drives persistent changes in histone modification patterns, nucleosome positioning, and higher-order chromatin organization, enabling the storage of inflammatory signal history (26, 66, 94, 95) (**Table 1**). The integration of these layers determines whether immune exposure induces reversible displacement within the identity landscape or promotes transition toward alternative stabilized states characterized by reduced functional competence (41, 96–98). Importantly, this framework permits quantitative interpretation of immune effects in terms of signal strength, duration, and combinatorial context, establishing a mechanistic basis for graded modulation of cellular identity rather than binary state transitions (99, 100). By explicitly delineating these hierarchical control points, this Perspective advances a structured model that links immune signaling dynamics to durable alterations in cellular state and provides a foundation for experimentally testable predictions regarding identity stability and reversibility.

## Cellular identity as a dynamic, immune-sensitive state

Cellular identity is often treated as a stable endpoint established by developmental programs and maintained thereafter by lineage-defining transcriptional networks (101, 102). Instead, increasing evidence supports a model in which differentiated identity is continuously maintained and therefore intrinsically dynamic, with cells occupying a range of states that include fully differentiated functionally competent phenotypes as well as stress-adapted and dysfunctional configurations (103, 104). Transitions along this continuum need not imply irreversible fate conversion (105). Rather, they reflect context-dependent remodeling of gene regulatory circuits that preserve viability while recalibrating specialized function (106, 107). Within this framework, immune-derived signals emerge as potent determinants of state occupancy because they converge on transcriptional control nodes and chromatin regulatory machinery that sustain lineage fidelity.

Metabolic tissues are particularly susceptible to immune-mediated modulation of identity because their specialized functions impose sustained biosynthetic and secretory demands that amplify vulnerability to inflammatory stress (27, 104). Chronic nutrient excess, fluctuating energy availability, and persistent activation of stress-response pathways create a background in which even modest inflammatory inputs can destabilize the transcriptional architecture required for mature function (105). In parallel, the low-grade inflammation characteristic of metabolic disease provides continuous exposure to cytokines, chemokines, and interferon-linked programs that can suppress lineage-defining genes, activate alternative transcriptional modules, and reinforce maladaptive states through epigenetic remodeling (108, 109). Thus, in metabolic organs immune signals are positioned to influence cellular identity, not episodically, but as a persistent pressure shaping the balance between functional specialization and stress adaptation (110).

At a systems level, maintenance of cellular identity requires continuous stabilization of gene regulatory networks against intrinsic noise and extrinsic perturbation (111, 112). This stability is achieved through tightly coupled feedback between lineage-defining transcription factors, enhancer landscapes, and metabolic state, which together constrain cells within a defined region of transcriptional state space (113, 114). Immune-derived signals perturb this equilibrium by introducing context-dependent regulatory inputs that alter both network connectivity and signal propagation dynamics (115, 116). Cytokine-induced activation of signaling pathways such as NF-κB and STAT does not simply superimpose new transcriptional outputs but reweighs existing regulatory interactions by modifying transcription factor binding kinetics, altering enhancer-promoter coupling, and redistributing limiting cofactors across competing gene programs (117, 118). These effects are further modulated by cell-type-specific chromatin accessibility landscapes, which determine the susceptibility of particular loci to immune-mediated regulation (119). As a result, the impact of immune signaling on identity is inherently non-uniform and depends on the pre-existing regulatory configuration of the cell (120, 121). This framework explains how equivalent inflammatory inputs can produce divergent outcomes across tissues, ranging from transient adaptive shifts in functional output to progressive destabilization of lineage fidelity (122, 123). By explicitly incorporating network stability, regulatory competition, and chromatin context, this model provides a mechanistic basis for understanding cellular identity as a dynamically maintained and quantitatively tunable state under immune control (124, 125).

The endocrine pancreas provides a tractable and informative model for studying immune regulation of cellular identity (126–128). Endocrine cell types are exceptionally specialized and depend on tightly constrained transcriptional programs to sustain stimulus secretion coupling and hormone production (129). These programs are highly sensitive to inflammatory cues that perturb calcium handling, endoplasmic reticulum homeostasis, and mitochondrial metabolism, thereby exposing points of fragility in the networks that enforce identity (130, 131). Importantly, functional impairment in the endocrine pancreas commonly precedes substantial cell loss, suggesting that immune and inflammatory pathways can drive clinically meaningful failure through state remodeling rather than destruction (10, 11, 53). For these reasons, the endocrine pancreas offers a clear setting in which to interrogate how immune signals modulate transcriptional and epigenetic mechanisms of identity maintenance and how such modulation contributes to durable dysfunction.

## Pattern recognition and toll-like receptor signaling as upstream determinants of identity modulation

Toll-like receptors (TLRs) function as spatially distributed molecular sensors that detect pathogen-associated and damage-associated signals (18, 132, 133). Rather than acting solely as initiators of inflammatory cascades, TLRs encode environmental information into quantitatively and temporally structured signaling outputs that are interpreted in a cell-type-specific manner. Ligand recognition by TLRs initiates activation programs across immune and non-immune compartments (19, 134, 135). These programs coordinate epithelial barrier function, macrophage positioning, stromal activation, and tissue-level inflammatory tone. Upon ligand recognition, TLRs assemble adaptor-specific signaling complexes. These complexes are organized around myeloid differentiation primary response protein 88 (MyD88) or TIR domain-containing adaptor inducing interferon beta (TRIF) (20, 89, 136, 137). They couple receptor activation to nuclear factor kappa B (NF-κB), interferon regulatory factors (IRFs), and mitogen-activated protein kinase (MAPK) pathways (138). The resulting signaling architecture produces graded and non-linear outputs defined by variation in amplitude, duration, and oscillatory dynamics, rather than binary activation states. Signal propagation is further shaped by ligand composition, receptor localization, and prior exposure history, establishing a context-dependent signaling logic that integrates environmental inputs into stable cellular responses (48, 56, 139, 140). In tissue contexts, TLR-dependent signaling extends beyond cytokine production and directly regulates functional gene expression programs. This is demonstrated in epithelial repair systems, hepatic inflammatory responses, stromal differentiation pathways, and neuroimmune circuits, where TLR activation modifies cell behavior, tissue organization, and local immune niches in ways that are tightly coupled to physiological state (141–144).

With sustained or repetitive stimulation, TLR signaling transitions from transient activation to chromatin-level regulation. It reshapes nucleosome organization and alters chromatin accessibility. It also stabilizes enhancer usage at inflammatory and stress-responsive loci (21, 22, 26, 51, 145). These chromatin-level effects are reinforced by signal-dependent transcription factors such as NF-κB and IRFs, whose activation dynamics encode information about stimulus duration and intensity. Repeated engagement of these factors drives the formation of inducible enhancers that sustain gene expression programs even after signal withdrawal (21). In metabolic and chronically stressed tissues, these mechanisms are particularly consequential because sustained TLR signaling integrates with nutrient excess, mitochondrial dysfunction, and cytokine exposure to establish low-grade inflammatory circuits. These circuits reprogram cellular metabolism and progressively destabilize lineage-specific transcriptional networks while preserving cell viability (10, 146). Taken together, these findings suggest TLR signaling as an upstream and integrative regulator of cellular identity, whereby environmental cues are translated into durable transcriptional and epigenetic states. In this framework, TLR-driven programs may modulate the balance between adaptive plasticity and progressive functional decline across tissues.

## Cytokine-driven fate instability: from lineage fidelity to identity drift

Cytokine-driven fate instability refers to a progressive reduction in the stability of lineage-defining gene regulatory networks induced by sustained inflammatory signaling, resulting in impaired functional output without necessitating loss of cellular viability (8, 10) (**Figure 1**). In differentiated tissues, lineage fidelity depends on the continuous reinforcement of transcription factor occupancy, enhancer activity, and chromatin accessibility at identity-defining loci (147). Persistent exposure to pro-inflammatory cytokines perturbs this equilibrium by introducing competing regulatory inputs that alter network connectivity and transcriptional resource allocation (148–150). Rather than inducing discrete fate transitions, cytokines shift cells within a constrained state space, producing graded deviations from the fully differentiated attractor state (23, 151). This process provides a mechanistic framework linking chronic inflammation to durable tissue dysfunction through immune-instructed remodeling of cellular state (152, 153).

### Destabilization of lineage regulatory networks

Differentiated cellular identity is sustained by interconnected gene regulatory networks anchored by lineage-defining transcription factors that couple specialized functions to stable chromatin states (35, 154). Pro-inflammatory cytokines can erode this stability by engaging signaling pathways that intersect directly with transcriptional machinery and its epigenetic scaffolding (155). At a mechanistic level, cytokine signaling pathways, including JAK–STAT, NF-κB, and interferon-responsive networks, modulate the balance between lineage-enforcing and stress-responsive transcriptional programs through dynamic redistribution of transcription factor binding and cofactor availability (29, 156). Through coordinated effects on transcription factor abundance, localization, and activity, inflammatory inputs weaken the circuits that maintain lineage fidelity and reduce occupancy at key regulatory elements (157–159). This redistribution alters enhancer utilization and promoter engagement, leading to selective attenuation of genes associated with specialized function while promoting activation of alternative transcriptional modules (30, 160). In parallel, cytokine signaling activates stress response programs and inducible transcriptional modules that compete with lineage networks for limiting cofactors and chromatin accessibility, thereby shifting the regulatory balance away from mature identity. Importantly, these effects arise not only from direct transcriptional repression but from competitive interactions between regulatory programs operating under limiting cofactor conditions (30, 156).

### Progressive identity drift rather than binary fate switching

A consistent consequence of cytokine exposure is the suppression of mature functional gene programs coupled with the emergence of alternative transcriptional states that resemble progenitor-like or partially-reprogrammed configurations (67, 130). This process is not adequately described by models that frame cell fate decisions as discrete switches between stable endpoints (27). Instead, cytokines promote identity drift, defined here as a partial and progressive erosion of lineage-specific transcriptional programs without complete loss of lineage affiliation. Cells retain core lineage markers yet exhibit diminished expression of functional effectors and regulatory factors required for specialized activity, while acquiring stress-associated or developmentally-permissive signatures (161, 162). This state reflects a reconfiguration of the underlying gene regulatory network rather than a binary fate transition, with cells occupying intermediate positions along a continuum defined by regulatory stability and functional competence (30, 163). Inflammatory remodeling of enhancer landscapes and promoter usage can further reinforce this drift, stabilizing altered regulatory states and constraining re-engagement of lineage-specific programs without necessitating overt lineage conversion (27, 164–166).

### Functional consequences of cytokine-induced identity drift

The implications of this framework are that inflammatory cytokines can generate populations of cells that remain viable but are functionally compromised and transcriptionally altered in ways that are intrinsically self-stabilizing (24, 167). Such cells may retain residual lineage markers while failing to execute specialized tasks, thereby contributing to tissue dysfunction in the absence of substantial cell loss (67, 168, 169). These altered states can become self-reinforcing through feedback between transcriptional and epigenetic mechanisms, sustaining dysfunction even in the absence of ongoing high-intensity inflammatory signaling (163, 170). Recognizing cytokine-driven identity drift as a primary pathogenic mechanism provides a mechanistic basis for understanding how chronic inflammation produces durable tissue dysfunction through progressive destabilization of cellular identity rather than direct cytotoxicity (24, 27).

## Metabolic tissues as sensitive readouts of immune control of cellular identity

Metabolic tissues occupy a unique position at the interface of immune signaling and cellular identity as their specialized functions must be continuously sustained under conditions of fluctuating environmental and physiological demand (171, 172). This requirement for persistent functional reinforcement places differentiated states under constant regulatory pressure, rendering them particularly sensitive to immune-mediated perturbation and permissive to remodeling of transcriptional and epigenetic programs (171, 173–175). As a result, metabolic organs provide a highly informative context in which to examine how immune-derived signals influence the stability and adaptability of cellular identity during chronic stress and inflammation.

This heightened sensitivity arises from the intrinsic coupling of lineage-specific transcriptional programs to cellular bioenergetic state (37, 176). In metabolically active tissues, maintenance of specialized function depends on sustained coordination between mitochondrial oxidative capacity, redox balance, and substrate flux, thereby directly linking gene regulatory stability to metabolic resource availability (177–179). Immune signaling perturbs this coupling by inducing coordinated reprogramming of intracellular metabolic pathways, including alterations in glycolytic throughput, tricarboxylic acid cycle activity, and lipid metabolism, which collectively reshape the energetic and biosynthetic environment that constrains transcriptional output (180–182). Concurrently, changes in metabolite availability modulate the activity of chromatin-regulatory enzymes whose catalytic functions depend on cofactors such as acetyl-CoA, α-ketoglutarate, and NAD^+^, thereby translating immune-induced metabolic shifts into locus-specific alterations in chromatin accessibility and histone modification states (183–185). Through these integrated effects, immune signals impose selective pressure on gene regulatory networks, preferentially destabilizing energetically demanding lineage-defining programs while permitting the emergence of transcriptional configurations compatible with reduced functional output (186–188). As metabolic tissues operate near the limits of biosynthetic and secretory capacity, even modest inflammatory inputs can induce disproportionate disruptions in regulatory equilibrium, providing a mechanistic basis for the early onset of functional impairment in the absence of overt cell loss (189–191).

### Persistent physiological stress as a driver of identity vulnerability

Metabolic tissues provide a uniquely informative context in which to define how immune signals regulate cellular identity as they operate under persistent physiological pressures that continuously test the stability of differentiated state programs (192). Unlike organs, in which specialized function is intermittent, hepatocytes, adipocytes, myocytes, and endocrine cells are chronically exposed to fluctuating nutrient availability and sustained energetic demand (42, 193). Continuous nutrient flux is coupled to elevated mitochondrial activity and reactive oxygen species production, establishing a baseline of oxidative stress that sensitizes transcriptional, epigenetic, and proteostatic networks to disruption (194–196). Superimposed on this metabolic environment is the low-grade inflammation associated with obesity, insulin resistance, and related cardiometabolic states, resulting in prolonged exposure to cytokines, chemokines, and interferon-linked signaling (197). Under these conditions, immune-derived cues act not as transient perturbations but as sustained inputs that shape the transcriptional and epigenetic configurations required for mature cellular function (173, 198).

### The endocrine pancreas as a paradigmatic case study

The endocrine pancreas exemplifies the vulnerability of metabolic tissues to immune-mediated identity modulation with particular clarity (130, 199). Endocrine cell types operate within a tightly constrained functional regime in which transcriptional precision, stimulus–secretion coupling, and metabolic flux must remain highly synchronized to sustain hormone output (200). This degree of specialization imposes stringent regulatory constraints on gene expression programs, rendering them acutely sensitive to perturbation by inflammatory signals that disrupt calcium handling, endoplasmic reticulum homeostasis, and mitochondrial function (199, 201). These stress-responsive pathways intersect directly with identity-enforcing transcriptional networks, exposing critical points of fragility in the maintenance of differentiated state (202, 203).

At a mechanistic level, immune-derived cytokines reconfigure β-cell regulatory architecture by altering transcription factor occupancy, enhancer activity, and chromatin accessibility at loci governing insulin biosynthesis, glucose sensing, and secretory machinery (204–206). These changes are reinforced by concurrent metabolic perturbations, including mitochondrial dysfunction and oxidative stress, which further constrain the energetic capacity required to sustain high-level insulin production (207–209). The convergence of transcriptional interference and metabolic limitation shifts regulatory equilibrium away from a fully differentiated state toward stress-adapted configurations that retain viability but exhibit impaired functional output (210, 211).

Importantly, functional impairment in the endocrine pancreas consistently precedes substantial cell loss, indicating that immune and inflammatory pathways drive clinically relevant dysfunction primarily through progressive destabilization of cellular identity rather than direct cytotoxicity (162, 212–214). This temporal dissociation provides a uniquely tractable system for resolving early-stage identity perturbations before irreversible structural damage occurs (209, 215, 216). As such, the endocrine pancreas serves not only as an example of immune-mediated dysfunction but as a model system in which the dynamics of identity stability, plasticity, and failure can be interrogated with high mechanistic resolution (210, 215, 217, 218).

### Generalizable principles of immune regulated identity

More broadly, metabolic tissues reveal immune control of cellular identity with exceptional resolution because their function depends on continuous reinforcement of differentiated programs and because disease states are often characterized by durable yet potentially reversible shifts in cellular state (193). These features make metabolic organs powerful systems for dissecting how chronic inflammatory signaling intersects with gene regulatory architecture to bias cells toward adaptive or maladaptive identities. Insights gained from these tissues are therefore likely to extend beyond metabolism, informing general principles by which immune signals shape tissue function through transcriptional and epigenetic reprogramming rather than cell elimination (171).

## Immune-epigenetic coupling: how inflammation becomes durable

Inflammatory signals frequently outlast their initiating stimuli by becoming encoded within the epigenetic structure of target cells. Through this process, immune-mediated cues are translated into stable modifications of chromatin and gene regulatory potential, allowing transient immune activation to produce sustained effects on cellular identity and function. Immune epigenetic coupling therefore represents a central mechanism by which inflammation acquires durability and exerts long-term influence on tissue state.

### Epigenetic remodeling as a mechanism of immune instruction

Immune signals exert long-lasting effects on cellular identity by directly reshaping the epigenetic architecture that governs transcriptional potential (27, 219). Inflammatory cytokines and interferon-linked pathways engage chromatin-modifying enzymes and transcriptional cofactors that alter chromatin accessibility at lineage-defining and stress-responsive loci (28, 130). This convergence is mediated by signal-activated transcription factors that function as sequence-specific targeting platforms, directing chromatin-modifying activities to defined genomic regions and thereby ensuring spatial precision in the epigenetic response to immune stimulation (220, 221). These changes include redistribution of open chromatin regions, persistent modification of histone marks associated with active or repressed states, and context-dependent alterations in DNA methylation (67, 131, 222). Through these mechanisms immune signals convert transient extracellular cues into durable intracellular configurations that stabilize altered transcriptional programs even in the absence of continued immune stimulation (223, 224). Collectively, these processes establish a mechanistic bridge between extracellular inflammatory cues and intracellular regulatory architecture by translating transient signaling events into structured chromatin modifications (225, 226).

Beyond local chromatin modifications, immune signaling exerts higher-order effects on genome organization (31, 227). Cytokine-induced signaling pathways can alter three-dimensional chromatin topology by modulating long-range interactions between promoters and distal regulatory elements, including enhancers and super-enhancers (202, 203). These changes influence the spatial configuration of the genome within the nucleus, thereby reshaping the regulatory context in which gene expression occurs (228, 229). Through reorganization of chromatin looping and compartmentalization, immune signals can selectively reinforce or disrupt regulatory hubs that coordinate expression of functionally related gene sets (32, 230). Such spatial reconfiguration extends the impact of immune signaling beyond individual loci to coordinated gene networks, amplifying its effects on cellular state without requiring widespread genomic disruption (231, 232).

Epigenetic modifications function in an integrated manner to redefine the probability landscape for transcription factor binding and transcriptional initiation across the genome (33, 233). In this context, epigenetic remodeling can be understood as altering the distribution of accessible versus inaccessible regulatory elements, thereby biasing transcriptional outcomes toward specific gene expression programs (34, 234). This redefinition reflects a shift in regulatory accessibility in which certain loci become preferentially permissive to transcriptional activation while others are progressively restricted, effectively biasing gene expression programs without requiring direct genetic change (33, 235, 236). Importantly, this process operates through modulation of binding site availability, cofactor recruitment efficiency, and enhancer activity, thereby influencing both the magnitude and stability of transcriptional responses (237–239).

Through these mechanisms immune signals convert transient extracellular cues into durable regulatory configurations capable of maintaining altered transcriptional states independent of continued upstream stimulation, thereby stabilizing changes in cellular identity over time (26, 240). The persistence of these configurations is supported by reinforcing feedback mechanisms, including sustained histone modification patterns, continued occupancy of key regulatory elements, and maintenance of chromatin accessibility states across cell divisions (241, 242). Such feedback enables propagation of the altered epigenetic landscape in the absence of ongoing signaling input, effectively encoding prior immune exposure into the regulatory state of the cell (243, 244). This capacity for self-maintenance distinguishes epigenetic remodeling as a mechanism not only of immediate transcriptional regulation but also of long-term stabilization of altered cellular identity (245–247).

### Transcription factor–epigenetic integration as a core mechanism of immune instruction

A mechanistic understanding of immune–epigenetic coupling requires explicit integration of transcription factors as central intermediaries that translate extracellular immune signals into durable chromatin states (246, 248, 249). While epigenetic remodeling is often described in terms of histone modifications, chromatin accessibility, and DNA methylation, these processes are not autonomous. Rather, they are orchestrated by signal-responsive transcription factors that interpret immune-derived cues and direct epigenetic machinery to specific genomic loci (250, 251).

Immune-activated transcription factors, including nuclear factor kappa B (NF-κB), signal transducer and activator of transcription (STAT) family members, and interferon regulatory factors (IRFs), function as primary determinants of how inflammatory signals are encoded within the genome (162, 252, 253). Upon activation, these factors exhibit highly dynamic binding behavior characterized by stimulus-specific kinetics, cooperative interactions, and context-dependent genomic occupancy (254, 255). Through sequence-specific DNA binding and interaction with cofactors, they recruit chromatin-modifying complexes such as histone acetyltransferases (for example, p300/CBP), histone deacetylases, methyltransferases, and ATP-dependent chromatin remodelers (256–258). This recruitment establishes or reinforces regulatory elements, including promoters and enhancers, that define transcriptional competence (257, 259).

A key feature of this process is the formation and stabilization of inducible enhancers. Signal-dependent transcription factors can establish *de novo* enhancer regions or amplify the activity of pre-existing regulatory elements, leading to sustained transcriptional outputs even after the initiating immune stimulus has diminished (260–263). These enhancers are often marked by increased chromatin accessibility, enrichment of activating histone modifications such as H3K27ac, and recruitment of mediator complexes that facilitate transcriptional initiation and elongation. In this manner, transient immune signals are converted into persistent regulatory states that reshape gene expression programs over extended timescales (264–266).

Importantly, transcription factors do not act in isolation but function within combinatorial regulatory networks (267, 268). The integration of multiple signaling pathways, such as cytokine-driven JAK–STAT signaling, NF-κB activation, and interferon responses, results in cooperative or competitive binding at shared genomic loci (269, 270). This combinatorial control determines the specificity, magnitude, and durability of transcriptional responses (271, 272). In inflammatory contexts, such interactions can shift the balance between lineage-defining transcription factors and stress-responsive factors, thereby destabilizing identity-enforcing gene regulatory networks (273–275).

This dynamic interplay has direct consequences for cellular identity. Lineage-defining transcription factors rely on stable chromatin accessibility at key regulatory regions to maintain differentiated function. Immune-activated transcription factors can disrupt this stability by redistributing cofactor availability, altering enhancer usage, and modifying higher-order chromatin organization (276, 277). As a result, lineage-specific transcriptional programs are attenuated, while alternative or stress-associated programs are reinforced (278, 279). Over time, repeated or sustained immune signaling can lead to progressive erosion of lineage fidelity and the emergence of hybrid or dysfunctional transcriptional states (279–281).

Temporal dynamics are critical in determining the outcome of transcription factor–mediated epigenetic remodeling (280–282). Acute activation of immune-responsive transcription factors may induce reversible changes in chromatin accessibility that support adaptive responses to stress (25, 283). In contrast, chronic or repetitive activation promotes the stabilization of these changes through epigenetic reinforcement mechanisms, including persistent histone modification patterns, altered nucleosome positioning, and changes in three-dimensional genome organization (284, 285). These stabilized states can become self-sustaining and reduce dependence on continued upstream signaling, thereby contributing to the persistence of dysfunctional cellular identities (286, 287).

Furthermore, transcription factors serve as potential regulatory checkpoints that govern the transition between plastic and fixed identity states (38, 288). The threshold, duration, and combinatorial activity of transcription factor networks may determine whether immune-induced changes remain reversible or become epigenetically locked. Disruption of these regulatory nodes may therefore represent a critical step in the progression from adaptive plasticity to pathological identity fixation (289, 290).

Integrating transcription factor dynamics into the framework of immune-regulated cellular identity provides a more complete mechanistic model of how immune signals shape tissue function (39, 291). It establishes transcription factors not merely as downstream effectors of signaling pathways but as active architects of the epigenetic landscape (292, 293). This perspective also provides a conceptual bridge between transient immune activation and long-term alterations in cellular state, thereby explaining how inflammation can exert durable effects on tissue function without necessarily inducing cell death (39, 294, 295).

### Epigenetic immune memory beyond the immune system

A critical implication of immune-driven epigenetic remodeling is the emergence of epigenetic immune memory in non-immune cells (131, 296). Analogous to trained immunity in innate immune populations, parenchymal cells exposed to inflammatory environments retain molecular imprints that influence future responses and constrain identity trajectories (157, 297, 298). These imprints are established through stable alterations in enhancer accessibility, histone modification patterns, and chromatin organization at regulatory regions associated with inflammatory and stress-responsive gene programs. The epigenetic imprints bias gene regulatory networks toward stress-adapted or dysfunctional states by lowering the threshold for reactivation of inflammatory programs and limiting reengagement of lineage-enforcing transcriptional circuits (127, 299).

Mechanistically, this bias reflects persistent changes in transcription factor accessibility and cofactor recruitment dynamics, which favor rapid reactivation of previously engaged regulatory elements while restricting access to loci required for fully differentiated function (300–302). As a result, immune exposure history becomes embedded within the epigenetic landscape of tissues, shaping cellular behavior over extended timescales (303, 304). This embedded memory does not simply reflect prior activation but actively constrains the range of permissible future cellular states, thereby influencing how cells respond to subsequent environmental or inflammatory challenges (305, 306).

### Consequences for persistent tissue dysfunction

Immune epigenetic coupling provides a mechanistic explanation for the persistence of tissue dysfunction after overt inflammation has been resolved (27). By stabilizing maladaptive transcriptional states through chromatin-based mechanisms, immune signals decouple cellular identity from immediate environmental conditions and lock cells into configurations that impair specialized functions (130, 154, 307). The stabilization is mediated by sustained occupancy of regulatory elements, maintenance of permissive or repressive chromatin marks, and reinforcement of transcriptional programs through epigenetically encoded feedback loops that persist in the absence of active signaling (308, 309). This framework suggests that chronic disease reflects not only ongoing immune activity but also the enduring legacy of prior inflammatory encounters encoded within tissue epigenomes (310). Importantly, the persistence of these states can lead to functional rigidity, in which cells exhibit reduced capacity to re-enter fully differentiated programs despite removal of the initiating inflammatory stimulus. Recognizing immune epigenetic memory as a determinant of tissue identity has important implications for therapeutic strategies, indicating that restoration of function may require targeted reversal of epigenetic constraints rather than the suppression of inflammation alone (28, 311). Such approaches may necessitate precise modulation of chromatin accessibility, enhancer activity, or transcription factor engagement to re-establish lineage-specific regulatory networks and restore functional competence.

## Dedifferentiation reconsidered: pathology or immune-imposed adaptation

Dedifferentiation in inflamed tissues has traditionally been viewed as a hallmark of irreversible failure, yet emerging evidence indicates that immune signals can actively instruct transient relaxation of differentiated programs as a means of stress adaptation (126, 312). This perspective raises the possibility that dedifferentiation reflects a regulated and context-dependent response to immune pressure rather than a uniform pathological endpoint.

### Reframing dedifferentiation in inflammatory contexts

Dedifferentiation is commonly interpreted as a pathological regression reflecting loss of lineage identity and irreversible failure of specialized function (27, 127). Within inflammatory disease settings, this interpretation has reinforced the view that immune activity primarily drives cellular damage (307). However, accumulating evidence suggests that dedifferentiation may instead represent an adaptive response to immune-mediated stress in which cells transiently relax differentiated programs to preserve viability under adverse conditions (129, 130). This relaxation involves selective reduction of lineage-specific gene expression rather than complete loss of identity. In this context, immune signals act not solely as injurious stimuli but as instructive cues that promote reversible identity modulation aimed at maintaining cellular integrity when full functional output is unsustainable (27, 101, 313). Such modulation reduces the energetic and regulatory burden associated with sustaining highly specialized functions during prolonged stress.

At a mechanistic level, inflammatory signaling alters transcription factor engagement and regulatory element usage at loci governing specialized function, leading to a controlled decrease in transcriptional output (314, 315). This shift preserves core cellular programs while limiting processes that impose high metabolic or biosynthetic demand (162, 316). As a result, cells enter a state characterized by reduced functional activity but maintained viability (317). This state can be viewed as a stress-adapted configuration that prioritizes survival over maximal functional performance (318, 319). Importantly, maintenance of partial lineage identity distinguishes this process from terminal fate loss and enables potential recovery upon resolution of inflammatory pressure (320, 321).

### A temporal model of immune driven identity modulation

A useful framework for reconciling adaptive and pathological interpretations of dedifferentiation is to consider the temporal dynamics of immune signaling (27, 126). Acute immune activation can induce partial and potentially reversible shifts in transcriptional state, reducing metabolic burden and limiting susceptibility to stress-induced damage (12, 128, 322). These early changes involve transient reduction in lineage-specific transcriptional output while preserving core regulatory architecture. Such adaptive plasticity may confer short-term protection by suppressing energetically costly lineage functions while preserving core identity features (130, 323). During this phase, regulatory elements associated with specialized function remain accessible, allowing rapid reactivation once inflammatory signals decline.

In contrast, chronic or recurrent immune signaling can progressively stabilize these altered states through epigenetic reinforcement, leading to fixation of dysfunctional identities that no longer support specialized function (10, 223). Prolonged signaling alters regulatory element accessibility and sustains engagement of stress-associated transcriptional programs, limiting reactivation of lineage-specific gene expression (91, 324). This shift reflects a transition from transient regulatory adjustment to stable reconfiguration of transcriptional control (325). Under these conditions, dedifferentiation transitions from a protective response to a pathogenic endpoint (326, 327). The duration and intensity of immune exposure therefore determine whether identity modulation remains reversible or becomes functionally restrictive (128, 328).

### Distinguishing plasticity from fixation

The critical distinction between adaptive plasticity and pathological fixation lies in the reversibility of immune-induced state changes (329). Plastic responses retain the capacity to reengage lineage-enforcing transcriptional programs once inflammatory pressure is relieved, whereas fixed states are maintained by durable alterations in chromatin organization and transcription factor networks that resist reversion (27, 131). Plastic states preserve accessibility at lineage-specific regulatory elements and maintain the underlying transcriptional framework required for functional recovery (221, 330). In contrast, fixed states exhibit restricted access to lineage-associated loci and sustained activation of alternative regulatory programs, thereby limiting restoration of specialized function (331, 332).

At a mechanistic level, plasticity is characterized by transient shifts in transcription factor engagement and regulatory element usage that remain responsive to changes in the extracellular environment (331, 333). Fixed states, however, are reinforced by stable alterations in regulatory element accessibility and sustained bias toward stress-associated transcriptional activity, which diminish responsiveness to upstream signaling cues (334). Recognizing this distinction reframes dedifferentiation as a spectrum of immune-instructed identity modulation rather than a uniform marker of failure and highlights the importance of defining the molecular thresholds at which adaptive responses become irreversibly maladaptive (8, 130). Identification of these thresholds is essential for determining when intervention can restore functional identity rather than merely limit further decline (276, 335).

### Reversibility and the concept of an identity point of no return

A central question in immune-mediated tissue dysfunction is whether altered cellular identities can be restored or whether inflammatory exposure ultimately drives cells beyond a threshold of recoverability (27, 126). The concept of an identity point of no return captures the idea that immune-driven state changes may transition from reversible adaptations to permanently stabilized dysfunction depending on the duration and intensity of inflammatory signaling (307, 312). Reversibility depends on the preservation of lineage-specific regulatory element accessibility and the capacity to reestablish transcription factor engagement at key identity-defining loci. Cells that retain this regulatory competence can restore functional gene expression once inflammatory pressure is relieved.

Progression toward an irreversible state is associated with sustained restriction of lineage-associated regulatory regions and persistent activation of alternative transcriptional programs (25, 281). These changes reduce the ability of cells to respond to environmental cues that would otherwise promote recovery (170, 336). The transition to a point of no return therefore reflects a shift from a flexible regulatory state to one that is stably maintained and resistant to reprogramming (282, 283). The duration and intensity of immune exposure determine whether identity modulation remains reversible or becomes permanently fixed (277, 280). Defining this threshold has important implications for therapeutic intervention, as restoration of function is most feasible before stable regulatory constraints are fully established (278, 280).

### Evidence for immune modulated plasticity

Accumulating evidence indicates that immune-imposed alterations in cellular identity are not uniformly irreversible and that restoration of function is possible under defined conditions. Attenuation or removal of inflammatory stress can permit reactivation of lineage-defining transcriptional programs and recovery of specialized functions, even in cells that have undergone substantial identity remodeling (24, 127, 337). This recovery depends on retention of accessible regulatory elements at key lineage-associated loci. These observations support the existence of a plastic phase in which immune-driven state changes remain labile and responsive to changes in the inflammatory milieu (174, 338). In this phase, regulatory element accessibility and transcription factor binding potential are preserved, enabling rapid re-engagement of lineage programs.

Transcriptional networks retain sufficient integrity to reestablish lineage fidelity once immune pressure is relieved or modulated (162, 221). During this phase, regulatory element accessibility and transcription factor binding potential are preserved, enabling rapid re-engagement of lineage programs. Reversibility is therefore determined by the capacity of cells to reassemble functional transcriptional circuits rather than by the extent of prior perturbation alone (131, 279, 280). Cells that maintain this capacity can return to a fully differentiated state with restoration of functional output (281, 282). Progressive loss of regulatory accessibility limits this recovery potential and promotes transition toward more stable dysfunctional states (335, 339).

## Temporal limits of identity reversibility

Despite this plasticity, immune-driven identity changes do not appear to remain indefinitely reversible (340, 341). Persistent inflammatory signaling can progressively stabilize altered cellular states through reinforcement of epigenetic constraints that limit access to lineage-enforcing regulatory elements (3, 28, 130). This stabilization reflects sustained restriction of regulatory element accessibility and reduced capacity for transcription factor re-engagement at key loci. As these constraints accumulate, cells may cross a threshold beyond which reengagement of differentiated programs becomes inefficient or impossible, defining an identity point of no return (222, 342). Crossing this threshold marks a transition from a responsive regulatory state to one that is resistant to reprogramming. The molecular features that distinguish reversible from fixed states remain incompletely defined but are likely to involve specific combinations of chromatin modifications, transcription factor network disruption, and alterations in higher order genome organization (27, 126). Defining these features will be essential for identifying early indicators of irreversible identity stabilization.

### Outstanding questions and therapeutic implications

Critical questions remain regarding which epigenetic modifications are amenable to reversal, how long immune-imposed identity states can persist before fixation occurs, and whether tissues possess discrete windows of recoverability determined by the duration or intensity of immune exposure (127, 147, 343). A central challenge is to define the hierarchy of regulatory elements that govern reversibility, including the relative contributions of promoters, enhancers, and higher-order chromatin interactions. It remains unclear whether loss of accessibility at a limited subset of key regulatory loci is sufficient to constrain recovery or whether broader network-level disruption is required. Resolving these issues has direct clinical relevance because it suggests that therapeutic efficacy may depend not only on the molecular target, but also on the timing of intervention relative to immune-driven state transitions (2).

An additional unresolved question concerns the kinetics of identity stabilization during sustained immune exposure (344, 345). Early stages may involve reversible redistribution of transcription factor binding and transient shifts in regulatory element usage, whereas prolonged exposure is likely to induce stable restriction of lineage-associated loci and reinforcement of alternative transcriptional programs (346, 347). Defining the temporal sequence of these events will be essential for identifying inflection points at which reversibility is lost. Interventions applied during periods of residual plasticity may enable restoration of identity and function, whereas delayed treatment may be constrained by epigenetically stabilized dysfunction (222, 348, 349).

Therapeutic development must therefore integrate both molecular specificity and regulatory state awareness (350, 351). Targeting upstream inflammatory pathways may be effective in early stages but insufficient once regulatory constraints are established at the level of chromatin accessibility and transcriptional control (352, 353). Restoration of function may require strategies that directly reconfigure regulatory architecture, including reactivation of lineage-associated loci, modulation of enhancer activity, and re-establishment of transcription factor networks. Such approaches will need to account for cell-type-specific regulatory landscapes and the extent of prior identity disruption (354, 355).

A key translational priority is the identification of biomarkers that report on the reversibility of cellular states *in vivo* (356, 357). These may include measures of regulatory element accessibility, transcription factor occupancy, or expression signatures that reflect underlying regulatory competence (357, 358). Integration of these parameters with temporal information on immune exposure could enable stratification of patients based on recoverability and guide stage-specific therapeutic intervention (359, 360). Advancing this framework will require coordinated analysis of chromatin state, transcriptional dynamics, and functional output across disease progression (361, 362).

## Extending the framework beyond the pancreas

The principles outlined in the endocrine pancreas reflect a broader mode of immune-tissue interaction that is not organ specific but instead emerges wherever the differentiated function must be actively maintained under conditions of chronic physiological stress (27). Viewing immune signaling as a regulator of cellular identity rather than a trigger of damage provides a lens through which diverse patterns of tissue dysfunction can be understood within a unified framework (67, 363).

### Immune regulation of identity across metabolic tissues

Across metabolic organs, immune-derived cues intersect with lineage specific transcriptional programs to reshape cellular state in ways that preserve viability while compromising specialized function (8, 10, 101). This intersection reflects targeted modulation of regulatory elements controlling metabolic and biosynthetic gene expression rather than global disruption of cellular identity. In the liver, inflammatory signaling modifies hepatocyte identity by altering gene expression, suppressing metabolic specialization, and reinforcing stress-responsive transcriptional states that persist after inflammation resolves (322, 364, 365). These changes involve reduced expression of genes governing lipid and glucose metabolism alongside sustained activation of inflammatory transcriptional regulators. In adipose tissue, cytokines released by resident and infiltrating immune cells drive remodeling of adipocyte identity, promoting inflammatory and fibrotic programs while constraining lipid storage and endocrine activity (110, 149, 173). This remodeling is accompanied by altered transcriptional control of adipogenic regulators and persistent activation of pathways linked to extracellular matrix deposition.

In skeletal muscle, sustained cytokine exposure induces metabolic redefinition characterized by impaired mitochondrial efficiency and altered substrate utilization, thereby uncoupling contractile identity from metabolic capacity (153, 366). This uncoupling reflects disruption of transcriptional coordination between energy production pathways and contractile gene programs. In each case, immune signaling produces durable shifts in identity without necessitating overt lineage conversion (367–369). These shifts arise from selective reweighting of gene regulatory networks that favor stress adaptation over specialized function while maintaining core lineage markers (370). The convergence of these processes across tissues indicates a shared principle in which immune signaling modulates functional output through regulatory reconfiguration rather than structural loss of cell identity (371, 372).

### A generalizable organizing principle of tissue immunology

These cross-tissue observations support the concept that immune regulation of cellular identity constitutes a core organizing principle of tissue immunology (173). This principle positions immune signaling as a continuous regulator of cellular state rather than a transient response to injury or infection. Immune systems continuously engage parenchymal cells to tune functional output in response to environmental and physiological stress, thereby shaping tissue behavior over time (373, 374). Such engagement occurs through context-dependent modulation of gene regulatory networks that determine the balance between specialized function and stress adaptation. When this regulatory interaction becomes chronic or dysregulated, immune-instructed identity shifts can become stabilized and pathogenic (110, 368). Under these conditions, regulatory adjustments that are initially adaptive may transition into persistent alterations that constrain functional capacity.

Framing tissue dysfunction through this lens integrates immune signaling with transcriptional and epigenetic control of identity as well as providing a common conceptual foundation for understanding chronic disease across organ systems (368, 369). This framework unifies diverse pathological states by linking immune activity to sustained changes in regulatory architecture rather than isolated tissue-specific defects (375, 376). It further suggests that variation in disease manifestation reflects differences in how individual tissues integrate immune-derived signals within their intrinsic regulatory networks (377, 378). By defining immune regulation of identity as a central organizing axis, this perspective enables cross-organ comparison and provides a basis for identifying shared mechanisms of dysfunction despite distinct physiological contexts (379, 380).

## Implications for immunology as a discipline

The recognition that immune systems actively regulate cellular identity rather than merely enforce defense and tolerance necessitates a substantial expansion of the conceptual foundations of immunology (28, 129, 381). Traditional models have emphasized immune-mediated elimination of pathogens and damaged cells, with tissue pathology viewed largely as collateral injury or failure of immune restraint (109, 199). However, accumulating evidence across tissues indicates that immune signals operate continuously as interpreters of physiological stress and environmental change, translating these cues into regulatory inputs that shape transcriptional and epigenetic programs in parenchymal cells (382–385). From this perspective, immune activity emerges as a determinant of tissue state over time, influencing how differentiated identities are maintained, adapted, or destabilized under chronic stress (108).

### Immune systems as organizers of tissue identity and state

Immune pathways function as active organizers of tissue identity by modulating the stability of lineage-defining gene regulatory networks (108, 109, 195). This organizational role reflects the capacity of immune signals to influence both the strength and persistence of regulatory interactions that maintain differentiated states. Through sustained cytokine signaling and cell-cell interactions, immune systems influence chromatin accessibility, transcription factor activity, and epigenetic memory in non-immune cells, thereby calibrating functional output to prevailing physiological conditions (28, 384). Such modulation occurs through selective regulation of key genomic loci that control specialized functions, allowing dynamic adjustment of transcriptional output without disrupting core lineage identity. This regulatory role enables tissues to adapt to transient challenges by modulating specialized functions while preserving viability (28, 174, 307). Adaptive modulation therefore represents a controlled rebalancing of regulatory network activity in response to environmental stress.

When immune signaling becomes prolonged or dysregulated, these adaptive adjustments can be reinforced into stable but maladaptive identity states that compromise tissue performance (171, 384). This reinforcement involves sustained alteration of regulatory element accessibility and persistent engagement of stress-associated transcriptional programs (386, 387). Over time, these changes reduce the capacity of cells to reestablish lineage-specific gene expression patterns, even after normalization of external conditions (388, 389). Viewing immune systems as regulators of identity and state therefore provides a framework for understanding how chronic inflammation leads to functional decline without extensive cell loss (118, 121). This perspective shifts emphasis from cell loss to regulatory dysfunction as the primary determinant of tissue impairment (390, 391). It further highlights immune signaling as a central integrator of environmental inputs that continuously shapes tissue identity across physiological and pathological contexts (117, 392).

### Integrating immunology with epigenetics, identity theory, and systems biology

Considering immune regulation of identity at the center of tissue biology creates a natural convergence between immunology and complementary disciplines (127). This convergence reflects the shared focus of these fields on mechanisms that govern stability, adaptability, and constraint within cellular systems. Immune-mediated remodeling of chromatin and transcriptional networks directly intersects with epigenetic mechanisms that encode cellular memory and constrain fate potential (28, 393). Such intersections occur through coordinated regulation of chromatin accessibility, transcription factor engagement, and regulatory element activity, linking immune signaling to durable changes in gene expression capacity. At the same time, the graded and context-dependent effects of immune signals on identity align with systems biology concepts of network stability, attractor states, and state transitions (338, 394, 395). In this context, immune signals function as modulators of network position within a defined state space rather than as binary determinants of cellular fate.

Integrating these perspectives allows immune signals to be understood not as isolated triggers, but as components of dynamic regulatory systems that shape tissue behavior across molecular, cellular, and temporal scales (338, 393). This framework emphasizes the importance of network interactions, feedback dynamics, and regulatory thresholds in determining cellular responses to immune input. This integrative framework is essential for explaining why similar inflammatory stimuli can produce distinct outcomes depending on cellular context history and timing. Differences in prior regulatory states, chromatin configuration, and transcription factor availability create context-specific responses to identical signals. As a result, immune-mediated identity modulation reflects the interaction between external signaling and intrinsic regulatory architecture, rather than a uniform response to inflammation.

### Rethinking immune injury through the lens of immune instruction

This expanded view compels a reassessment of the long-standing dichotomy between immune-mediated damage and intrinsic tissue failure (338, 396). This reinterpretation shifts the focus from structural damage to regulatory reconfiguration as one of the primary drivers of dysfunction. Many pathological features traditionally attributed to immune injury may instead reflect immune-instructed remodeling of cellular identity that preserves survival while attenuating specialized function (396, 397). Such remodeling represents an active and regulated process rather than passive degeneration (349, 398). It involves coordinated modulation of transcription factor networks, chromatin accessibility, and regulatory element activity that reshape functional output without necessitating cell loss. Distinguishing immune instruction from immune destruction has profound implications for experimental interpretation, disease classification, and therapeutic design (108). It requires re-evaluation of phenotypes previously attributed solely to cytotoxic mechanisms in light of reversible or semi-stable identity changes.

This shifts emphasis from suppressing immune activity toward understanding how immune signals shape identity and how maladaptive identity states might be reversed (27, 399, 400). The perspective further highlights the need to define regulatory mechanisms governing transitions between adaptive modulation and stable dysfunction. By reframing immune tissue interactions in this way, immunology can more fully account for chronic disease mechanisms and uncover new strategies aimed at restoring tissue function through reestablishment of appropriate cellular state rather than elimination of immune responses (67, 298). Such strategies may include targeted restoration of lineage-defining regulatory programs, modulation of chromatin accessibility, and rebalancing of transcriptional networks. Collectively, this framework positions immune instruction as a central determinant of tissue pathology and a critical focus for therapeutic development.

## Future directions

### Defining immune-induced identity states

A central priority for the field is the systematic classification of immune-induced cellular identity states across tissues and disease contexts (67, 401, 402). While immune-mediated identity modulation is increasingly recognized, these states are often described in heterogeneous and tissue-specific terms that obscure shared principles (59). Developing a coherent taxonomy based on transcriptional, epigenetic, and functional features would enable comparison across organs and inflammatory settings. Such classification could reveal conserved immune-instructed identity programs and distinguish adaptive stress responses from maladaptive fixed states (403, 404). An improved framework for defining identity states is essential for integrating experimental findings and for establishing mechanistic links between immune signaling and durable tissue dysfunction (405).

### Identifying immune identity checkpoints

Another critical question is whether immune regulation of cellular identity is governed by discrete molecular checkpoints analogous to immune checkpoints that regulate lymphocyte activation (27). These identity checkpoints may consist of transcription factor nodes, chromatin modifiers, or signaling thresholds that determine whether immune-induced state changes remain reversible or become fixed (337). Defining such control points would provide insight into how immune signals are integrated over time and how cells transition between plastic and stabilized identity states (43, 397). Identifying identity checkpoints would also create opportunities for targeted intervention aimed at preserving or restoring differentiated functions without broadly suppressing immune activity.

### Predicting reversibility through immune epigenetic signatures

Determining whether immune epigenetic states can predict disease reversibility represents an important translational goal (406, 407). If specific chromatin configurations or transcriptional signatures mark the boundary between reversible and irreversible identity remodeling, they could serve as biomarkers to guide therapeutic timing and strategy. Establishing such predictors requires linking immune exposure history to epigenetic architecture and functional outcome at the single cell level (27, 36, 192). This approach would move the field beyond descriptive associations toward predictive models of tissue recovery and failure.

### Experimental strategies to advance the field

Addressing these questions will require experimental approaches that capture immune identity dynamics with high temporal and cellular resolution (24, 193). Longitudinal studies of human tissues are essential to define how immune signals shape identity over time and to distinguish transient adaptations from stable pathological states (171). Single-cell multiomics approaches that integrate transcriptomic, epigenetic, and immune-signaling data will be necessary to resolve heterogeneity and identify regulatory nodes underlying identity transitions (164). In parallel, temporal mapping of immune exposure and subsequent identity change will enable reconstruction of causal trajectories linking inflammation to durable tissue dysfunction (151). Together these strategies will provide the mechanistic foundation needed to translate the concept of immune regulated identity into predictive and therapeutic frameworks.

## Conclusions

The concepts advanced in this perspective converge on the recognition that immune regulation of cellular identity constitutes a fundamental and continuous axis of tissue control. Rather than acting exclusively through episodic activation that culminates in cytotoxicity or immune resolution, immune systems engage in sustained dialogue with parenchymal cells, interpreting metabolic, environmental, and inflammatory cues to shape transcriptional and epigenetic programs that define differentiated states (27, 408). Through this ongoing interaction, immune signals influence cellular function, adaptability, and vulnerability to failure, positioning immune instruction as a determinant of tissue behavior over time rather than a secondary consequence of pathology.

Metabolic disease exposes this regulatory function with particular clarity, as functional impairment frequently arises in the setting of chronic immune activation before extensive cell loss becomes apparent (409). However, the principles described are unlikely to be confined to metabolic tissues. Comparable patterns of immune-instructed identity modulation are increasingly evident across diverse organs in which specialized cellular programs must be actively maintained under physiological stress (396, 406). Viewed in this broader context, immune control of cellular identity represents a unifying mechanism through which inflammation produces durable alterations in tissue state and drives chronic disease progression (36, 171, 394).

Incorporating immune regulation of cellular identity into immunological theory necessitates a reassessment of how inflammation and tissue failure are conceptualized (127, 393). Immune-mediated pathology can be understood not solely as the result of destructive processes but as the outcome of persistent immune instruction that remodels the cellular state in adaptive or maladaptive directions (109). This perspective highlights the importance of temporal dynamics, context, and reversibility in shaping disease outcomes and suggests that effective therapeutic strategies may depend on restoring appropriate identity programs rather than simply attenuating immune activity.

## Data Availability

The original contributions presented in the study are included in the article/supplementary material. Further inquiries can be directed to the corresponding author.
